# Characterization of temperature-sensitive alleles of *Schizosaccharomyces pombe* septation initiation network components

**DOI:** 10.17912/micropub.biology.001249

**Published:** 2024-06-25

**Authors:** Lesley A. Turner, Alaina H. Willet, Kathleen L. Gould

**Affiliations:** 1 Department of Cell and Developmental Biology, Vanderbilt University School of Medicine, Nashville, TN, US

## Abstract

The *Schizosaccharomyces pombe* septation initiation network (SIN) promotes cytokinesis and septation. Comprised of a protein kinase cascade triggered by activation of a small GTPase and inhibited by a two-component GAP that localize to the spindle pole bodies in a cell cycle specific manner. Here, we characterized temperature-sensitive mutants isolated in the 1990s in four SIN components. We determined the mutations within each *cdc14* , *cdc16* , *sid1 ,* and *sid2* mutant allele and analyzed their growth at different temperatures compared with known mutant alleles. The new mutants described here expand the toolkit for studying SIN signaling.

**Figure 1. Characterization of sin mutant alleles.**
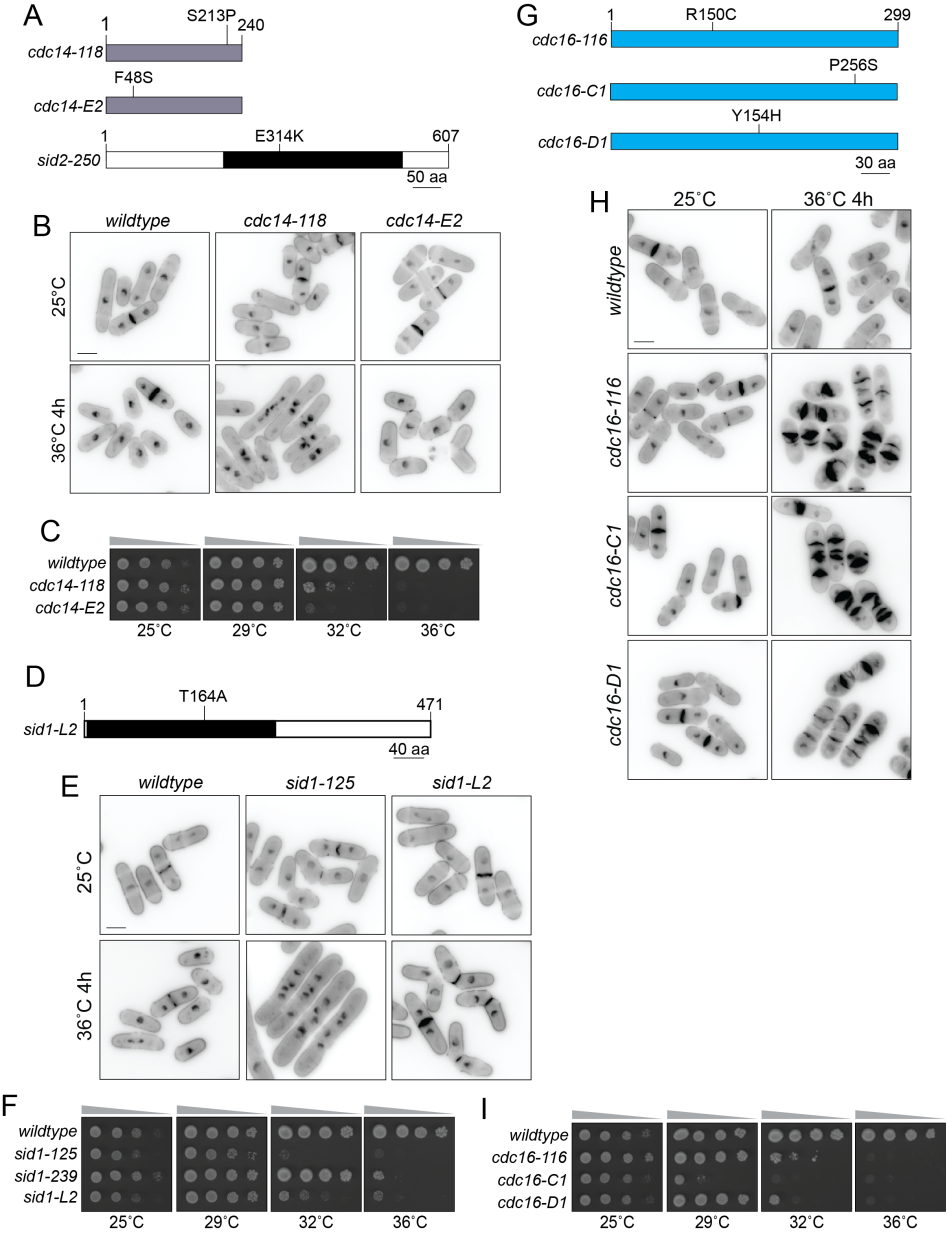
(A, D, and G) Schematics of Cdc14, Sid2, Sid1, and Cdc16, drawn to scale. The catalytic domains of Sid1 and Sid2 are indicated by the black boxes. The mutations encoded by the indicated temperature sensitive alleles are shown. (B, E, and H) The indicated strains were grown at 25˚C and shifted to 36˚C for 4 hours. Samples were collected at both temperatures and cells were fixed and stained with DAPI and methyl blue before imaging. Scale bar, 5 µm. (C, F, I) The indicated strains were grown in liquid YE at 25°C until they reached mid-log phase and then adjusted to the same cell concentration measured by optical density (Moreno et al., 1991). Then, 10-fold serial dilutions were made and 2.5 µL of each was spotted on YE agar plates and incubated at the indicated temperatures for 2-5 days prior to imaging.

## Description

Cell division in the yeast *Schizosaccharomyces pombe* requires a signaling cascade termed the septation initiation network (SIN) (reviewed in Cullati and Gould, 2019; Simanis, 2015; Xiao and Dong, 2021). The SIN is necessary for normal assembly, maintenance, and constriction of the actin- and myosin-based machinery required for cell division as well as activation of the cell wall enzymes necessary for septation (Cheffings et al., 2016; Glotzer, 2017; Mangione and Gould, 2019) . In *sin* mutants, cytokinesis fails and cells become elongated and multinucleate.

Activation of the SIN pathway is driven by the Spg1 GTPase (Schmidt et al., 1997; Sohrmann et al., 1998) that in turn activates the Cdc7 protein kinase (Fankhauser and Simanis, 1994; Schmidt et al., 1997) . The Sid1 kinase in complex with its cofactor Cdc14 acts next in the pathway followed by the Sid2 protein kinase in complex with its cofactor Mob1 (Fankhauser and Simanis, 1993; Guertin et al., 2000; Hou et al., 2000; Salimova et al., 2000; Sparks et al., 1999) . The Sid2- Mob1 complex is the only component of the SIN that localizes to the cell division site (Hou et al., 2000; Salimova et al., 2000; Sparks et al., 1999) . Byr4 and Cdc16 comprise a two-component GAP for Spg1 and are major SIN inhibitors (Furge et al., 1998; Minet et al., 1979) . SIN mutants have been isolated in genetic screens for general cell cycle regulators and also in those targeting cytokinesis factors (Balasubramanian et al., 1998; Nurse et al., 1976) . In several cases, the mutations within the mutant alleles have not been identified.

In addition to the *cdc14-118* allele described and characterized previously (Marks et al., 1992; Nurse et al., 1976) , we isolated a second mutant allele mapping to the *cdc14* gene, *cdc14-E2* (Balasubramanian et al., 1998) . The *cdc14* open reading frame (ORF) was amplified from each of the two strains and sequenced to determine what mutation was present. While the *cdc14-118* allele encoded a S213P substitution, the *cdc14-E2* allele encoded a F48S substitution ( Figure 1A ). To compare the cell phenotypes, we examined each mutant by staining for nuclei and septa after the cells were grown at 25°C and then shifted or not to 36˚C for 4 hours ( Figure 1B ). While *cdc14-118* cells showed the classic *sin* phenotype of multinucleation and cell elongation at the non-permissive temperature, *cdc14-E2* cells arrested uniformly at a very late stage of septation and frequently lysed ( Figure 1B ). A spot assay showed that the *cdc14-E2* allele was comparable in its temperature sensitivity to *cdc14-118* ( Figure 1C ).

Although the *sid2-250* mutant has been extensively analyzed (Balasubramanian et al., 1998) , the identity of the causative mutation has not been reported. The *sid2* open reading frame was therefore amplified from *sid2-250* cells, sequenced, and a single mutation was identified leading to a E314K substitution within the catalytic domain ( Figure 1A ).

In our screen for cytokinesis mutants, we identified several mutants that mapped to the *sid1* locus, one ( *sid1-L2* ) that has not been previously characterized, and two mutants mapping to the *cdc16* locus, *cdc16-C1* and *cdc16-D1* (Balasubramanian et al., 1998) . To determine if the *sid1-L2* allele differed from *sid1-125* (L114P) and *sid1-239* (L12P) *,* the *sid1* ORF was amplified from it and sequenced. A single point mutation causing a T164A substitution within the catalytic domain was found ( Figure 1D ). Nuclei and septa staining revealed predominantly a boomerang-shape phenotype that was often accompanied by cell lysis at septation ( Figure 1E ). A spot assay revealed that *sid1-L2* had an intermediate restrictive temperature compared to *sid1-125* and *sid1-239* ( Figure 1F ).

The *cdc16* ORFs were also amplified from *cdc16-116, cdc16-C1,* and *cdc16-D1* and sequenced to determine if the C1 and D1 alleles differed from *cdc16-116* . We found only single mutations in each ORF leading to three distinct amino acid substitutions ( Figure 1G ). To compare the cell phenotypes, we examined each mutant by staining for nuclei and septa after the cells were grown at 25°C and then shifted or not to 36˚C for 4 hours. The phenotypes of the three mutants were comparable. At 25˚C, the percent of septated cells was 17-20 with none showing more than one septa and at 36˚C, all cells arrested with multiple septa and one or two nuclei ( Figure 1H ). We next determined the range of temperature-sensitivity of each *cdc16* allele by spotting at a variety of temperatures. All temperature-sensitive alleles grew less than wildtype at 36°C with the *cdc16-C1* allele showing the greatest temperature-sensitivity ( Figure 1I ).

In sum, we have provided an initial characterization of new mutants of SIN components that expand the repertoire of reagents which can be used to study SIN signaling.

## Methods

Yeast methods

*S. pombe* strains were grown in yeast extract (YE) and standard *S. pombe* mating, sporulation, and tetrad dissection techniques were used to construct new strains (Moreno et al., 1991) . All spot assays were performed twice with reproducible results.

Molecular biology methods

*cdc14* alleles were amplified using an oligonucleotide 20 bp upstream of the start site (TATTGCCCGCTTGGCATGAG) and another 20 bp downstream of the stop codon (TAAGTTAACAATGAGACTTTAAACATT) (Integrated DNA technologies). *cdc16* alleles were amplified using an oligonucleotide 50 bp upstream of the start site (GAATTCATACTGGTCCTCATTTTAGT) and another 77 bp downstream of the stop codon (GAGTGAGAGGTGTGTGCTGA) (Integrated DNA technologies). The *sid2* allele was amplified using an oligonucleotide 70 bp upstream of the start site (ACACGTAAGGTTATTATTGACAGGAG) and another 50 bp downstream of the stop codon (CATCAAAAGCGAAGCTCAGTATCTTC) (Integrated DNA technologies). The PCR products were each sequenced by Plasmidsaurus (Eugene, OR) using Oxford Nanopore Technology with custom analysis and annotation. The *sid1-L2* open reading frame was amplified using an oligonucleotide 70 bp upstream of the start site (AGTACTTCGTGGTGCATCTAGCT) and another 70 bp downstream of the stop codon (GTAGAATATGCCATTATAAGTTCATT) (Integrated DNA Technologies). Sanger DNA sequencing was performed by GenHunter (Nashville, TN) using additional internal forward (GCGTGTCATCTTTGAAATTCCTCAATC) and reverse (CCAATACACTTTGATCCGAATCAT) primers (Integrated DNA technologies).

Microscopy and image analysis

Strains for fixed-cell imaging experiments were grown at 25°C in YE and then shifted to 36°C for 4 hours. Cells were fixed with 70% ethanol for DAPI and methyl blue (MB) staining as described previously (Roberts-Galbraith et al., 2009) . Images were acquired using a Zeiss Axio Observer inverted epifluorescence microscope with Zeiss 63× oil objective (1.46 NA) and captured using Zeiss ZEN 3.0 (Blue edition) software. A singular medial Z slice was obtained. All images were further processed using ImageJ (Schindelin et al., 2012) . All imaging experiments were repeated twice.

## Reagents

The strains used in this study and their genotypes are listed below.

**Strain** **Genotype** **Source**

KGY184 *cdc16-116 h ^-^ (Minet et al., 1979)*

KGY187 *cdc14-118 h ^-^ (Nurse et al., 1976)*

KGY246 *ade6-M210 leu1-32 ura4-D18* *h ^-^* Lab stock

KGY1052 *cdc14-E2 ura1 leu1-32 mam2::LEU2 ade6-M216 h ^90^ (Balasubramanian et al., 1998)*

KGY1055 *cdc16-D1 ura1 leu1-32 mam2::LEU2 ade6-M216 h ^90^ (Balasubramanian et al., 1998)*

KGY1057 *cdc16-C1 ura1 leu1-32 mam2::LEU2 ade6-M216 h ^90^ (Balasubramanian et al., 1998)*

KGY1168 *sid1-L2* *ade6-M21X ura4-D18 leu1-32 h ^-^* This study

KGY2089 *sid1-239 leu1-32 h ^-^* Lab stock

KGY4319 *sid1-125 ura4-D18 leu1-32 ade6-M21X h ^-^* Lab stock

KGY4875-2 *cdc16-C1 ade6-M21X ura4-D18 leu1-32 h ^-^* This study

KGY7146-2 *cdc16-D1 ade6-M21X leu1-32 ura4-D18 h ^-^* This study

KGY9160-2 *cdc14-E2 leu1-32 mam2::LEU2 ura4-D18 h ^90^* This study

KGY9560-2 *cdc14-E2 leu1-32 ura4-D18 h ^-^* This study

## References

[R1] Balasubramanian MK, McCollum D, Chang L, Wong KC, Naqvi NI, He X, Sazer S, Gould KL (1998). Isolation and characterization of new fission yeast cytokinesis mutants.. Genetics.

[R2] Cheffings TH, Burroughs NJ, Balasubramanian MK (2016). Actomyosin Ring Formation and Tension Generation in Eukaryotic Cytokinesis.. Curr Biol.

[R3] Cullati SN, Gould KL (2019). Spatiotemporal regulation of the Dma1-mediated mitotic checkpoint coordinates mitosis with cytokinesis.. Curr Genet.

[R4] Fankhauser C, Simanis V (1993). The Schizosaccharomyces pombe cdc14 gene is required for septum formation and can also inhibit nuclear division.. Mol Biol Cell.

[R5] Fankhauser C, Simanis V (1994). The cdc7 protein kinase is a dosage dependent regulator of septum formation in fission yeast.. EMBO J.

[R6] Furge KA, Wong K, Armstrong J, Balasubramanian M, Albright CF (1998). Byr4 and Cdc16 form a two-component GTPase-activating protein for the Spg1 GTPase that controls septation in fission yeast.. Curr Biol.

[R7] Glotzer M (2017). Cytokinesis in Metazoa and Fungi.. Cold Spring Harb Perspect Biol.

[R8] Guertin DA, Chang L, Irshad F, Gould KL, McCollum D (2000). The role of the sid1p kinase and cdc14p in regulating the onset of cytokinesis in fission yeast.. EMBO J.

[R9] Hou MC, Salek J, McCollum D (2000). Mob1p interacts with the Sid2p kinase and is required for cytokinesis in fission yeast.. Curr Biol.

[R10] Mangione MC, Gould KL (2019). Molecular form and function of the cytokinetic ring.. J Cell Sci.

[R11] Marks J, Fankhauser C, Simanis V (1992). Genetic interactions in the control of septation in Schizosaccharomyces pombe.. J Cell Sci.

[R12] Minet M, Nurse P, Thuriaux P, Mitchison JM (1979). Uncontrolled septation in a cell division cycle mutant of the fission yeast Schizosaccharomyces pombe.. J Bacteriol.

[R13] Moreno S, Klar A, Nurse P (1991). Molecular genetic analysis of fission yeast Schizosaccharomyces pombe.. Methods Enzymol.

[R14] Nurse P, Thuriaux P, Nasmyth K (1976). Genetic control of the cell division cycle in the fission yeast Schizosaccharomyces pombe.. Mol Gen Genet.

[R15] Roberts-Galbraith RH, Chen JS, Wang J, Gould KL (2009). The SH3 domains of two PCH family members cooperate in assembly of the Schizosaccharomyces pombe contractile ring.. J Cell Biol.

[R16] Salimova E, Sohrmann M, Fournier N, Simanis V (2000). The S. pombe orthologue of the S. cerevisiae mob1 gene is essential and functions in signalling the onset of septum formation.. J Cell Sci.

[R17] Schindelin J, Arganda-Carreras I, Frise E, Kaynig V, Longair M, Pietzsch T, Preibisch S, Rueden C, Saalfeld S, Schmid B, Tinevez JY, White DJ, Hartenstein V, Eliceiri K, Tomancak P, Cardona A (2012). Fiji: an open-source platform for biological-image analysis.. Nat Methods.

[R18] Schmidt S, Sohrmann M, Hofmann K, Woollard A, Simanis V (1997). The Spg1p GTPase is an essential, dosage-dependent inducer of septum formation in Schizosaccharomyces pombe.. Genes Dev.

[R19] Simanis V (2015). Pombe's thirteen - control of fission yeast cell division by the septation initiation network.. J Cell Sci.

[R20] Sparks CA, Morphew M, McCollum D (1999). Sid2p, a spindle pole body kinase that regulates the onset of cytokinesis.. J Cell Biol.

[R21] Sohrmann M, Schmidt S, Hagan I, Simanis V (1998). Asymmetric segregation on spindle poles of the Schizosaccharomyces pombe septum-inducing protein kinase Cdc7p.. Genes Dev.

[R22] Xiao Y, Dong J (2021). The Hippo Signaling Pathway in Cancer: A Cell Cycle Perspective.. Cancers (Basel).

